# LKB1 Regulates Goat Intramuscular Adipogenesis Through Focal Adhesion Pathway

**DOI:** 10.3389/fphys.2021.755598

**Published:** 2021-10-13

**Authors:** Yan Xiong, Yuxue Wang, Qing Xu, An Li, Yongqi Yue, Yan Ma, Yaqiu Lin

**Affiliations:** ^1^Key Laboratory of Qinghai-Tibetan Plateau Animal Genetic Resource Reservation and Utilization, Ministry of Education, Southwest Minzu University, Chengdu, China; ^2^Key Laboratory of Qinghai-Tibetan Plateau Animal Genetic Resource Reservation and Utilization, Sichuan Province, Southwest Minzu University, Chengdu, China; ^3^College of Animal and Veterinary Sciences, Southwest Minzu University, Chengdu, China; ^4^State Key Laboratory of Agrobiotechnology, China Agricultural University, Beijing, China

**Keywords:** IMF, intramuscular adipocyte, adipogenesis, LKB1, focal adhesion pathway, FAK, RNA-seq, RNA sequencing

## Abstract

Intramuscular fat (IMF) deposition is one of the most important factors to affect meat quality in livestock and induce insulin resistance and adverse metabolic phenotypes for humans. However, the key regulators involved in this process remain largely unknown. Although liver kinase B1 (LKB1) was reported to participate in the development of skeletal muscles and classical adipose tissues. Due to the specific autonomic location of intramuscular adipocytes, deposited between or within muscle bundles, the exact roles of LKB1 in IMF deposition need further verified. Here, we cloned the goat LKB1 coding sequence with 1,317 bp, encoding a 438 amino acid peptide. LKB1 was extensively expressed in detected tissues and displayed a trend from decline to rise during intramuscular adipogenesis. Functionally, knockdown of LKB1 by two individual siRNAs enhanced the intramuscular preadipocytes differentiation, accompanied by promoting lipid accumulation and inducing adipogenic transcriptional factors and triglyceride synthesis-related genes expression. Conversely, overexpression of LKB1 restrained these biological signatures. To further explore the mechanisms, the RNA-seq technique was performed to compare the difference between siLKB1 and the control group. There were 1,043 differential expression genes (DEGs) were screened, i.e., 425 upregulated genes and 618 downregulated genes in the siLKB1 group. The Kyoto Encyclopedia of Genes and Genomes (KEGG) analysis predicted that the DEGs were mainly enriched in the focal adhesion pathway and its classical downstream signal, the PI3K-Akt signaling pathway. Specifically, knockdown of LKB1 increased the mRNA level of focal adhesion kinase (FAK) and *vice versa* in LKB1-overexpressed cells, a key component of the activated focal adhesion pathway. Convincingly, blocking this pathway by a specific FAK inhibitor (PF573228) rescued the observed phenotypes in LKB1 knockdown adipocytes. In conclusion, LKB1 inhibited goat intramuscular adipogenesis through the focal adhesion pathway. This work expanded the genetic regulator networks of IMF deposition and provided theoretical support for improving human health and meat quality from the aspect of IMF deposition.

## Introduction

Intramuscular fat (IMF) was regarded as the most intramuscular adipocytes deposited between primary and secondary muscle bundles of perimysium, and a small population was also founded within muscle bundles (Li et al., 2020). The IMF accumulation was mainly determined by the hyperplasia and hypertrophy of intramuscular adipocytes, indicated by an increase in the number and size of adipocytes, respectively (Haczeyni et al., 2018). For farm animals, IMF was also referred to as marbling fat, which was highly desirable for improving the tenderness and flavor of meat quality (Hocquette et al., 2010; Hunt et al., 2016; Sun et al., 2019), while increasing IMF content in humans was associated with the occurrence of insulin resistance and adverse metabolic phenotypes, such as type 2 diabetes (Lim, 2014). Therefore, there are intensive efforts for elucidating the molecular mechanism of IMF deposition to enhance meat quality or defend against metabolic syndrome because of ectopic fat accumulation in skeletal muscle.

Liver kinase B1 (LKB1) also known as serine/threonine kinase 11 (STK11) is a serine/ threonine kinase (Berthelsen et al., 2021), which participates in the regulation of a variety of cellular physiological and pathological processes (Shan et al., 2016a,b; Xiong et al., 2017, 2018b) and phosphorylates 14 kinases of AMPK subfamily and regulates systemic glucose and energy balance (Lizcano et al., 2004). In skeletal muscle, LKB1 ablation exhibited a severe myopathy characterized by centrally nucleated myofibers, reduced mobility, growth retardation, and premature death (Shan et al., 2014), which is essential for the development and function of skeletal muscle (Shan et al., 2017). In classical adipose tissue, LKB1 gene knockout expanded the brown fat growth and increased mice energy expenditure and insulin sensitivity, associated with elevation of expression of thermogenesis-related genes (Shan et al., 2016b). Specifically, *MyoD-Cre-*derived Lkb1 deletion of muscle stem cells (satellite cells) and their descendent mature muscles increased lipid accumulation in proliferating myoblasts and myofibers (Shan et al., 2015). Intramuscular adipocyte was located within perimysium or muscle bundles, whose hyperplasia and hypertrophy were associated with reduced contractile function, insulin resistance, and type 2 diabetes (Li et al., 2020). Whether Lkb1 is involved in intramuscular adipocyte differentiation and lipid accumulation remains largely unclear. Additionally, the specific autonomic location and absence of maker genes of intramuscular adipocyte limited researchers to explore the molecular mechanism of its deposition *in vivo*.

Thus, the cultured intramuscular preadipocytes from the goat were used as the adipogenesis model to reveal the function and molecular mechanism of LKB1 regulation on IMF deposition. Here, the goat LKB1 gene-coding sequence was first cloned and further explored its function in intramuscular adipocytes by knockdown and overexpression techniques. Moreover, the RNA-seq was carried out to screen the differential transcripts affected by Lkb1 loss of function. Next, we provided sufficient evidence that LKB1 regulated adipogenesis of goat intramuscular preadipocytes through the focal adhesion pathway. Conclusively, these findings elucidated the specific molecular mechanism of LKB1 regulating the differentiation of intramuscular adipocytes and provided theoretical support for improving meat quality and human health.

## Materials and Methods

### Ethical Statement

All experimental procedures were reviewed and approved by the Institutional Animal Care and Use Committee, Southwest Minzu University (Chengdu). In addition, all the experiments complied with the requirements of the directory of the Ethical Treatment of Experimental Animals of China. The 7-day-old and 1-year-old Jianzhou Daer goats were reared by standard diet and housed in a comfortable environment. The goats were purchased from Sichuan Jianyang Dageda Animal Husbandry Co., Ltd. (Sichuan, China), and humanely slaughtered in the Key Laboratory of Qinghai-Tibetan Plateau Animal Genetic Resource Reservation and Utilization, Ministry of Education, Southwest Minzu University, Chengdu, China. The experimental animal certification number was SYXK-2019-216.

### Isolation and Adipogenic Differentiation of Primary Goat Intramuscular Preadipocytes

The isolation of primary goat intramuscular preadipocytes was carried out as previously reported (Xiong et al., 2018a; Huang et al., 2020). Briefly, longissimus dorsi was collected from slaughtered 7-day-old Jianzhou Daer goat after euthanatized by CO_2_. The samples were washed three times in phosphate-buffered solution (PBS) supplemented with 1% penicillin/streptomycin (P/S) and then were minced into a 1 mm^3^ cube. Enzymatic digestion dilution (DMEM/F12 with 2 mg/ml collagenase type II and 1% P/S) was used to isolate intramuscular preadipocytes at 37°C in the water bath for 1–1.5 h with gentle shaking and was terminated digestion by the same volume of DMEM/F12 (Hyclone, Logan, UT, USA) supplemented with 10% fetal bovine serum (FBS). The suspension was filtered through a 75-μm nylon cell strainer to remove connective tissues and undigested trunks of tissues. Cells then were centrifuged at 2,000 r/min for 5 min and re-suspended by red blood cell lysed solution for 30 min to lyse red blood cells. Next, the suspension was centrifuged at 2,000 r/min for 5 min again and the pre-adipocytes were resuspended in a growth medium (DMEM/F12 supplemented with 10% FBS and 1% P/S) and diluted to a final concentration of 10^6^ cells/ml. These cells were cultured at 37°C in a humidified atmosphere containing 5% CO_2_. It was previously reported that preadipocytes attached much earlier than myoblasts, the cultured cells were rinsed with PBS three times 1 h after plating to remove myoblasts, insoluble myofibrillar proteins, and other insoluble debris (Zhao et al., 2016; Sun et al., 2020). Cells were fed with fresh growth medium every 2 days until they reached 80% confluence and digested with 0.05% trypsin, then seeded at a density of 5 × 10^4^ cells/cm in a 6-well plate. The preadipocytes with 100% confluence were induced by induction medium (IM), containing 10% FBS, 1% P/S, and 50 μM of oleic acid (Sigma) and change the IM every 2 days. The time point of cells with 100% confluence was defined as day 0, and cell samples were collected at day 2 and day 4 post-induction.

### Adenovirus Generation and Infection

The adenovirus with goat LKB1 insertion was generated using the AdMax system as reported (Zhu et al., 2018). Briefly, the Coding Sequence (CDS) of LKB1 was cloned, inserted into the pHBAd plasmid, and verified by enzyme digestion and sequencing. The positive plasmid-containing goat LKB1 was named pHBAd-LKB1. Then, HEK293A cells (50–60% confluent) in 6 cm culture dishes were co-transfected 4 μg backbone plasmid (pBHGlox(delta) E1, 3Cre) with 2 μg pHBAd-LKB1 or 2 μg pHBAd empty plasmid using Lipofectamine 3000 (Life Technologies, Carlsbad, CA, USA) according to the protocol of the manufacturer. After 2 weeks, the two strains of recombinant adenovirus were collected by three freezes–thaw–vortex cycles, named LKB1OE and NC, respectively. Two more round infected HEK293A cells were adapted to amplify the recombinant virus, and the titers were determined by the expression of red fluorescence protein. Adenovirus purification by CsCl_2_ grade ultra-centrifuge based on described procedure (Mueller et al., 2012).

### Chemical Synthesis of siRNA and Transfection

Two individual siRNAs targeted for goat LKB1 were synthesized these sequences by Thermo Fisher Scientific. siRNA-1 (5′-CCAAGCUCAUCGGAAAGUACCUGAU-3′) and siRNA-2 (5′-GACAUUGAGGACGACGUCAUCUACA-3′). Negative control (NC) was provided by Invitrogen (5′-UUCUCCGAACGUGUCACGsUTT-3′). siRNA transfection was performed by Lipofectamine® RNAIMAX Reagent (Invitrogen, Waltham, MA, USA) at 70–80% preadipocytes confluence. Then, cells were analyzed by qPCR and oil red O staining at day 2 and day 4 after adipogenic induction.

### Oil Red O Staining

The oil red O staining was carried out as previously described (Xiong et al., 2018a). In brief, goat intramuscular adipocytes were washed with PBS two times and fixed with 4% formaldehyde for 15 min at room temperature. Then the cells were incubated using the oil red O working solution containing 6 ml oil red O stock solution (5 g/L in isopropanol) and 4 ml ddH_2_O for 30 min. After staining, the cells were washed with 60% isopropanol in PBS and pictured. Oil red O dye was extracted from stained adipocytes with 100% isopropanol, and the oil red O signal was quantified by measuring the optical density at 490 nm (OD 490).

### Bodipy Staining

The goat intramuscular adipocytes were removed from the IM and incubated by 2 μM of BODIPY™493/503 (Thermo Fisher Scientific, D3922) diluted in IM for 1 h. Then cells were washed with PBS three times, added fresh IM, and took pictures. Fluorescent images were captured using an Olympus TH4-200 microscope (Tokyo, Japan) with the 10× objective (NA 0.70) for higher magnification views. Images for control and treatment cells were captured using identical parameters.

### Total RNA Extraction and Quantitative Real-Time PCR (qPCR)

Total RNA was extracted from various tissues and intramuscular adipocytes using Trizol reagent (Takara) according to the protocol of the manufacturer. RNA was treated with RNase-free DNase I to remove genomic DNA. The purity and concentration of total RNA were measured by Nanodrop 3000 (Thermo Fisher). Ratios of absorption (260/280 nm) of all samples were between 1.8 and 2.0. Then 2 μg of total RNA were reversed transcribed using random primers and Moloney murine leukemia virus reverse transcriptase. qPCR was carried out with a Bio-Rad CFX96 PCR System using SYBR Green Master Mix (Takara) and gene-specific primers (Table S1). The 2^−Δ*ΔCT*^ method was used to analyze the relative changes of gene expression normalized against peptidylprolyl isomerase A (PPIA) as the internal control (Xiong et al., 2018a; Xu et al., 2018).

### Construction of RNA-Seq Library and Sequencing

A total amount of 3 μg RNA per sample was used as input material for the RNA sample preparations. First, ribosomal RNA was removed by Epicentre Ribo-zero™ rRNA Removal Kit (Epicentre, USA), and the rRNA-free residue was cleaned up by ethanol precipitation. Subsequently, sequencing libraries were generated using the rRNA depleted RNA by NEBNext® Ultra™ Directional RNA Library Prep Kit for Illumina® (NEB, Ipswich, MA, USA) following the recommendations of the manufacturer. Briefly, fragmentation was carried out using divalent cations under elevated temperature in NEBNext First Strand Synthesis Reaction Buffer (5×). First-strand cDNA was synthesized using random hexamer primer and M-MuLV Reverse Transcriptase (RNaseH-). Second strand cDNA synthesis was subsequently performed using DNA polymerase I and RNase H. In the reaction buffer, deoxynucleotide triphosphatesd (dNTPs) with deoxythymidine triphosphate (dTTP) were replaced by deoxyuridine triphosphate (dUTP). The remaining overhangs were converted into blunt ends via exonuclease/polymerase activities. After adenylation of 3′ ends of DNA fragments, NEBNext Adaptor with hairpin loop structure was ligated to prepare for hybridization. To select cDNA fragments of preferentially 150–200 bp in length, the library fragments were purified with the AMPure XP system (Beckman Coulter, Beverly, MA, USA). Then 3 μl USER Enzyme (NEB, Ipswich, MA, USA) was used with size-selected, adaptor-ligated cDNA at 37°C for 15 min followed by 5 min at 95°C before PCR. Then PCR was performed with Phusion High-Fidelity DNA polymerase, Universal PCR primers, and Index (X) Primer. At last, products were purified (AMPure XP system) and library quality was assessed on the Agilent Bioanalyzer 2100 system.

The clustering of the index-coded samples was performed on a cBot Cluster Generation System using TruSeq PE Cluster Kit v3-cBot-HS (Illumia) according to the instructions of the manufacturer. After cluster generation, the libraries were sequenced on an Illumina Hiseq 4000 platform and 150 bp paired-end reads were generated.

### DEG and KEGG Analysis

Cuffdiff provides statistical routines for determining differential expression in digital transcript or gene expression data using a model based on the negative binomial distribution (Trapnell et al., 2010). Transcripts with a *P-adjust*< *0.05* were assigned as differentially expressed. KEGG is a database resource for understanding high-level functions and utilities of the biological system (Kanehisa et al., 2008). We used KOBAS software to test the statistical enrichment of differential expression genes (DEGs) in KEGG pathways (Mao et al., 2005).

### MTT Assay

MTT (50 mg) was dissolved in 10 ml PBS (pH 7.2) to obtain a concentration of 5 mg/ml. The induced intramuscular adipocytes were seeded in 96-well plates with approximately 3,000 cells per well. Different final concentrations (0, 5, 10, 50, and 100 nM) of focal adhesion kinase (FAK) inhibitor (PF573228) were added into wells, respectively. After 24 and 48 h treated by FAK inhibitor, 10% MTT was added to wells to incubate for 4 h at 37°C. Then, the sediment was dissolved in dimethylsulfoxide (DMSO) and the absorbance was measured at 490 nm.

### Statistical Analysis

All the data are presented as means ± SEM. Comparisons were made by unpaired Student's *t*-test using SPSS 17.0 software (SPSS Science, Chicago, IL, USA). Effects were considered significant at *P*< *0.05*.

## Results

### Cloning and Bioinformatics Analysis of Goat *LKB1* Gene

**Figure 1:**
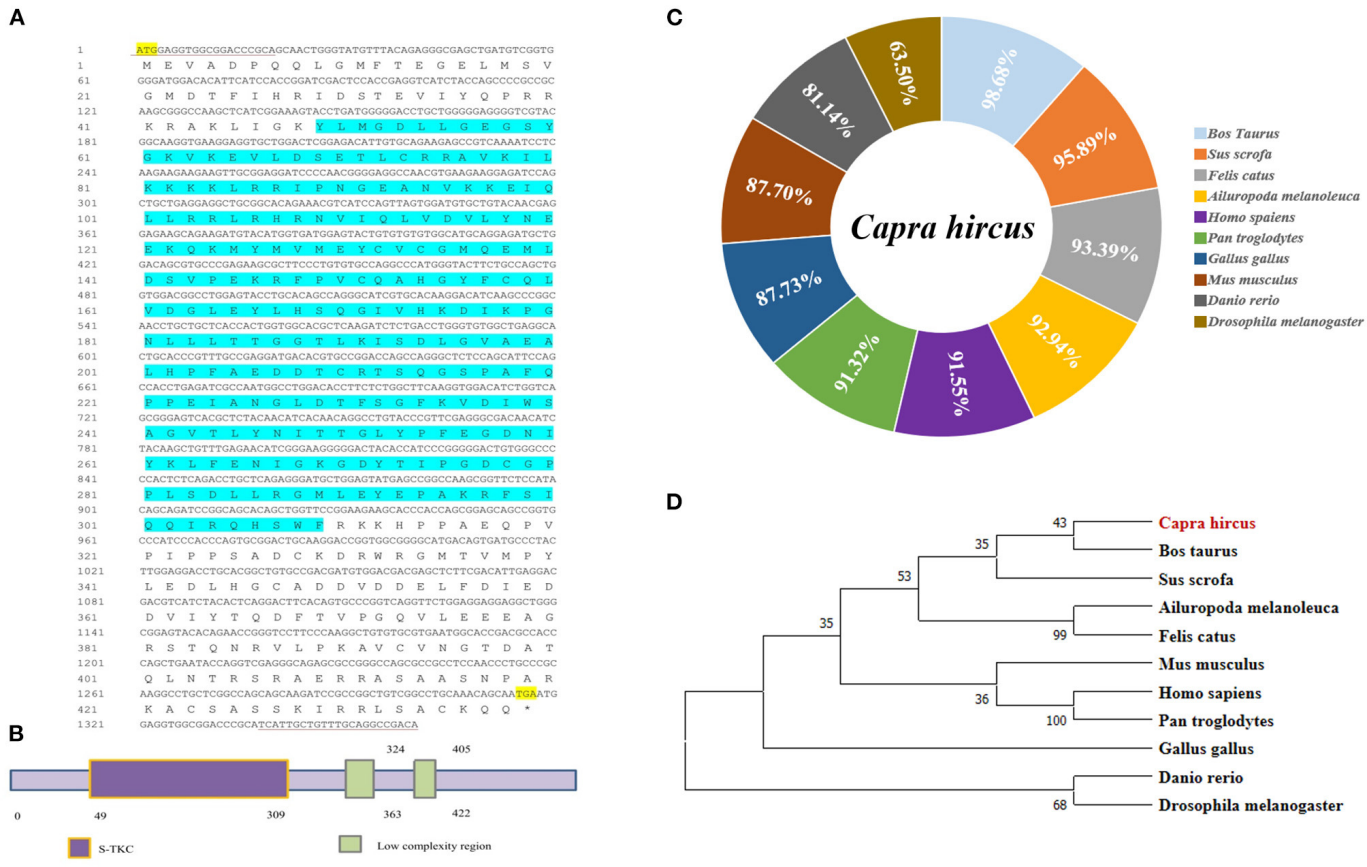
The cloning of goat LKB1 gene and its alignment analysis among species. **(A)** The nucleotide sequence and the translated amino acid sequence of goat *LKB1* (^*^ Represents the stop codon). **(B)** Prediction of the biological function of LKB1 amino acid sequence. **(C)** The amino acid sequence identity was analyzed by NCBI blast for LKB1 protein between goat and other mammalian species retrieved from GenBank. *Capra hircus* (MW664927), *Sus scrofa* (XP_020939922.1), *Ailuropoda melanoleuca* (XP_002923557.1), *Felis catus* (XP_003981653.1), *Homo sapiens* (NP_000446.1), *Pan troglodytes* (XP_024206909.1), *Gallus gallus* (NP_001039298.1), *Mus musculus* (NP_035622.1), Bos taurus (XP_024850893.1), *Danio rerio* (NP_001017839.1), and *Drosophila melanogaster* (NP_001163606.1). **(D)** LKB1 amino acid sequence phylogenetic tree was constructed by ClustalX 1.83 and MEGA7.0. LKB1, liver kinase B1.

As the loss of validated sequence of goat *LKB1* gene in the NCBI database, its mRNA sequence was first cloned to further elucidate its function in intramuscular adipogenesis. The data showed that the full length of the LKB1 gene was 1,380 bp and was cloned by PCR using cDNA of longissimus dorsi as a template, including 1,317 bp complete open reading frame (ORF) region sequence, encoding a 438 amino acid peptide, and 63 bp 3′ untranslated region (3′UTR) sequence (Figure 1A). Next, the protein functional prediction showed that goat LKB1 protein had a typical (serine/threonine kinases catalytic) S-TKC domain (Figure 1B). Then, the amino acid sequence homology of LKB1 protein between goat and other animals was analyzed by NCBI blast (Figure 1C). The results showed that goat LKB1 protein was highly identical to *Bos Taurus* (98.68%). Furthermore, the phylogenetic trees of LKB1 proteins, constructed by clustalx1.83 and mega5.0, showed that goat LKB1 protein had the closest relationship with those of cattle and pig, and the farthest relationship with that of *Danio rerio* and *Drosophila* (Figure 1D). These results suggest that the function of the LKB1 gene was relative conservation among species.

### The Expression Patterns of *LKB1* Gene in Goat Various Tissue and During Intramuscular Adipogenesis

**Figure 2:**
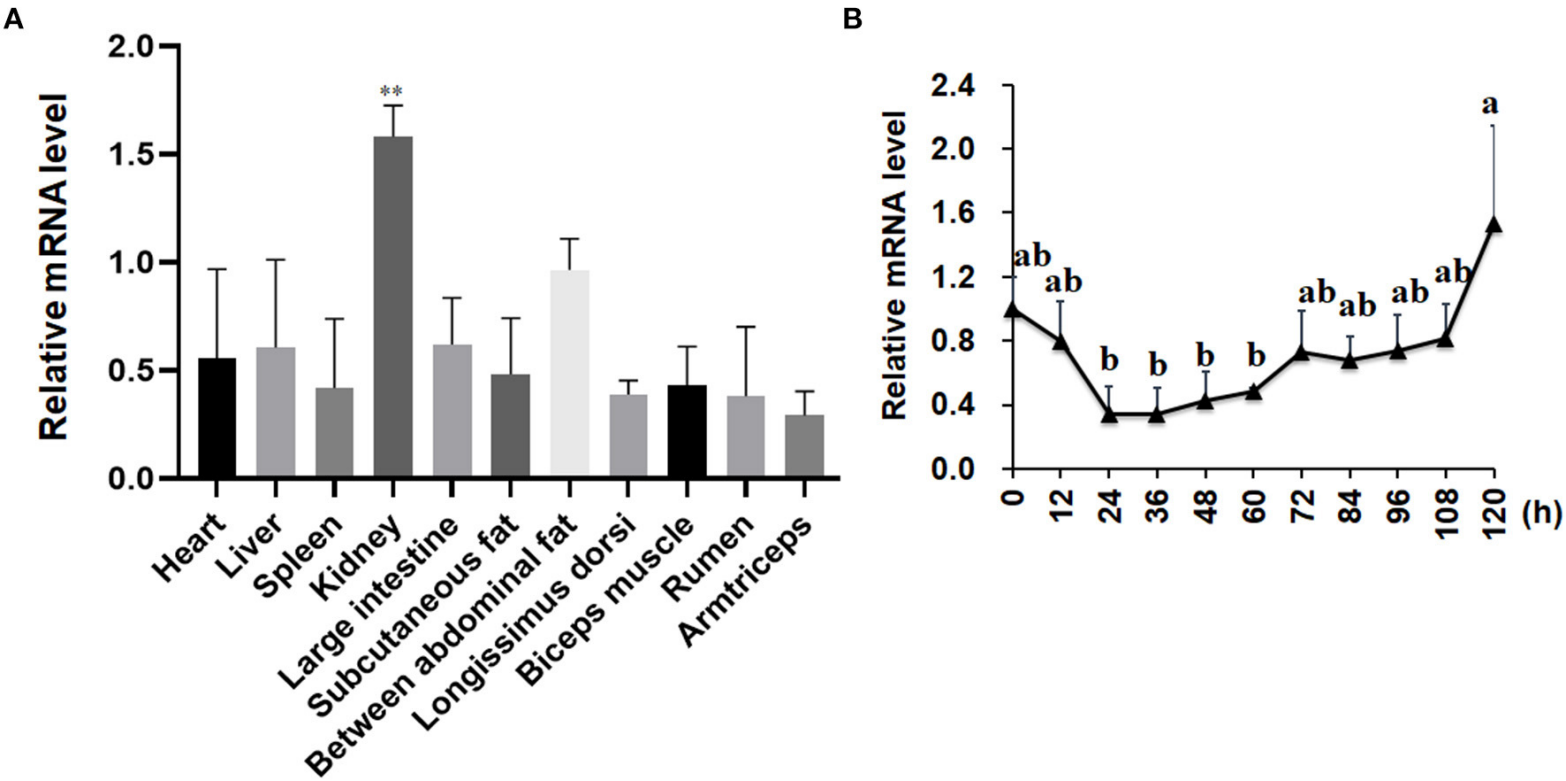
The mRNA expression patterns of the *LKB1* gene in goat tissues and during intramuscular adipocyte differentiation. **(A)** The *LKB1* mRNA level in heart, liver, spleen, kidney, large intestine, subcutaneous fat, between abdominal fat, longissimus dorsi, biceps muscle, rumen, and arm triceps, *n* = 6. Ribosomal protein lateral stalk subunit P0 (*RPLP0*) as the internal reference gene. **(B)** The *LKB1* mRNA level at day 0, 12, 24, 36, 48, 60, 72, 84, 96, 108, and 120 h in induced differentiation intramuscular adipocyte (*n* = 6), both *UXT* and *PPIB* are as reference genes. **P < *0.01*, the *LKB1* level of kidney compared to other tissues. Different letters indicate the significant difference (*P* < 0.05), and the same lowercase indicates an insignificant difference (*P* > 0.05). LKB1, liver kinase B1.

As shown in Figure 2A, the expression patterns of the LKB1 gene in goat various tissues, such as the heart, liver, spleen, kidney, large intestine, rumen, subcutaneous adipose tissue, abdominal adipose tissue, longissimus dorsi muscle, biceps femoris muscle, and triceps brachii muscle, were measured by qPCR. The data showed that LKB1 mRNA was widely expressed in detected tissues of goats, and the highest expression level was found in the kidney, which was significantly higher than that in other tissues (*P*< *0.01*). During intramuscular adipogenesis, the mRNA level of the LKB1 gene first displayed a declined trend, reaching the lowest point at 24 h post-induction, and then gradually increased in the late stage (Figure 2B). These results suggest that the *LKB1* gene might be related to IMF deposition in goats.

### Knockdown of *LKB1* Promotes Intramuscular Preadipocytes Differentiation

**Figure 3:**
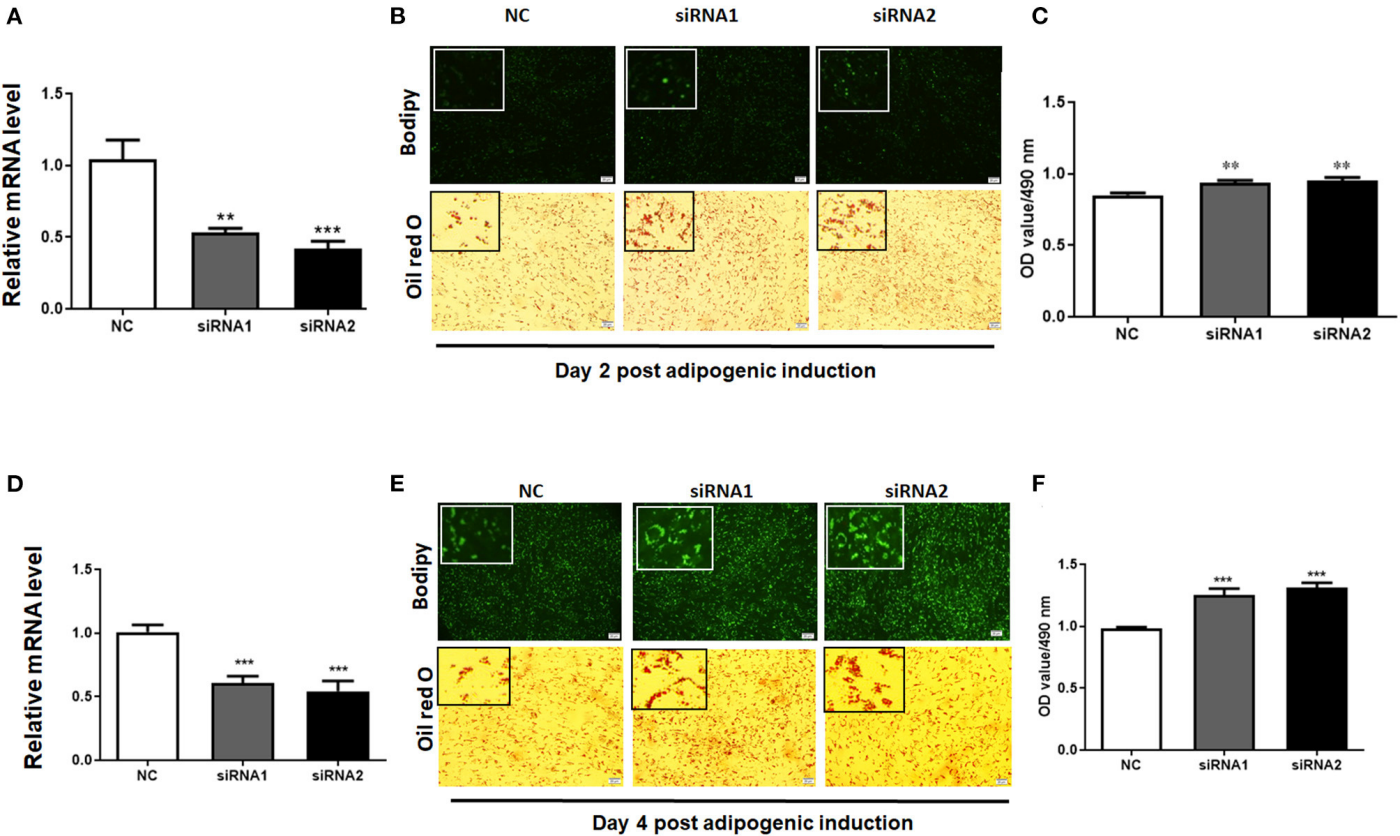
Knockdown of LKB1 promotes goat intramuscular adipocyte lipid accumulation. **(A,D)** The knockdown efficiency of LKB1 at day 2 **(A)** and day 4 **(D)** post-adipogenic induction (*n* = 6). **(B,E)** The bodipy and oil red O staining between control and siRNAs treatment intramuscular adipocyte cells. **(C,F)** The quantitative analysis of lipid accumulation in the negative control (NC) and siRNAs transfected groups was indicated by the OD value of oil red O dye at 490 nm at day 2 **(C)** and day 4 **(F)** after adipogenic induction (*n* = 6). **P < 0.01, ***P < 0.001, compared to that of NC. Scale bar: 100 μm. LKB1, liver kinase B1.

To elucidate the role of LKB1 in regulating intramuscular lipid accumulation in goats, two individual LKB1 siRNAs were transfected into goat intramuscular preadipocytes to perform loss of function. As shown in Figures 3A,D, these siRNAs significantly decreased the mRNA levels of LKB1 at day 2 and day 4 post-adipogenic induction, with knockdown efficiency of ~ 50% and ~ 60% to that of NC, respectively (*P*< *0.001*). At the morphology, bodipy staining showed that the LKB1 loss of function increased the bodipy dye in intramuscular adipocytes at day 2 adipogenic differentiation (Figure 3B). Consistently, oil red O staining also showed that knockdown of LKB1 promoted lipid droplet accumulation (Figure 3B). Statistically, LKB1 interference exhibited a dramatic increase in the signal of oil red O, indicated by the OD value at 490 nm (Figure 3C, *P*< *0.01*). On day 4 after adipogenic induction, knockdown of LKB1 also significantly increased both signals of bodipy and oil red O in intramuscular adipocytes (Figures 3D–F). These data suggest that loss of LKB1 function promotes lipid accumulation in goat intramuscular adipocytes.

**Figure 4:**
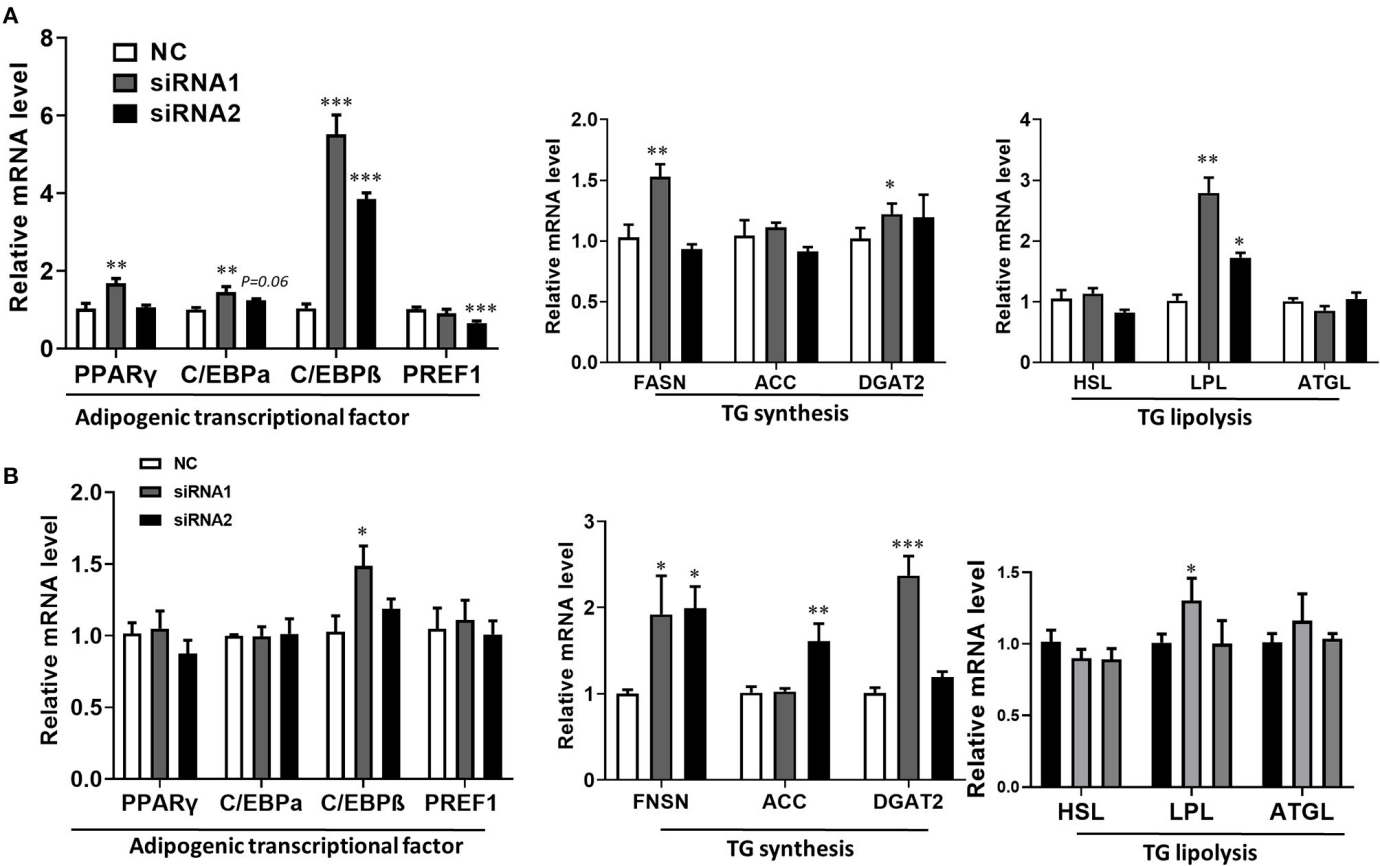
Knockdown of LKB1 promotes adipogenic and lipid accumulation-related genes. **(A,B)** The mRNA level of adipogenic transcription factors (*PPAR*γ, *C/EBP*α, *C/EBP*β, and *PREF1*), TG synthesis genes (*FASN, ACC*, and *DGAT2*), and TG lipolysis genes (*HSL, LPL, and ATGL*) between control and siRNAs groups at day 2 **(A)** and day 4 **(B)** post-adipogenic induction. *P < 0.05, **P < 0.01, ***P < 0.001, compared to that of control. FASN, fatty acid synthase; ACC, acetyl-CoA carboxylase alpha; DGAT2, diacylglycerol acyltransferase, HSL, hormone-sensitive lipase; LPL, lipoprotein lipase; ATGL, adipose triglyceride lipase.

The lipid droplets accumulation of adipocytes is a complex biological process, which is associated with preadipocyte initial adipogenic differentiation statue, triglyceride (TG) synthesis, and decomposition, summarized as adipocyte precursor cells differentiated into mature adipocytes (Gulyaeva et al., 2019). It was well known that adipogenesis is regulated by the central cascade transcription factors (CCAAT enhancer-binding proteins/peroxisome proliferator-activated receptor γ, C/EBPs/PPARγ), TG biogenic-related genes (FASN, fatty acid synthase; ACC, acetyl-CoAcarboxyla; DGAT2, diacylglycerol acyltransferase), and TG lipolytic-related genes (HSL, hormone-sensitive lipase; LPL, lipoprotein lipase; ATGL, adipose triglyceride lipase) (Engin, 2017). The qPCR analysis was carried out to determine the LKB1 loss of function effect on intramuscular adipogenesis at the molecular level. The data showed that siRNAs treatment significantly elevated the mRNA level of *PPAR*γ, *CEBP*α, and *CEBP*β. Specifically, interference of LKB1 respectively led to increasing by 5-fold and 4-fold changes of *CEBP*β in siRNA1 and siRNA2 transfected cells to that of NC at day 2 post-adipogenic induction (Figure 4A). Conversely, knockdown of LKB1 suppressed the mRNA level of delta-like non-canonical Notch ligand 1 (*DLK1 or PREF1*), a marker gene of preadipocytes (Figure 4A). Furthermore, the mRNA level of *FASN* and *DGAT2* was upregulated in siRNA1 treatment, while that of *ACC* was not significant between siRNAs and NC (Figure 4A). In addition, inhibition of LKB1 by siRNAs promoted the expression of lipolysis genes *LPL*, but not *HSL* and *ATGL* (Figure 4A). Consistently, similar trends of the aforementioned genes mRNA level were also observed at day 4 post-adipogenic induction, as shown in Figure 4B. Thus, these data indicated that LKB1 loss of function promotes goat intramuscular adipogenesis.

### Overexpression of *LKB1* Inhibits Intramuscular Adipogenesis

**Figure 5:**
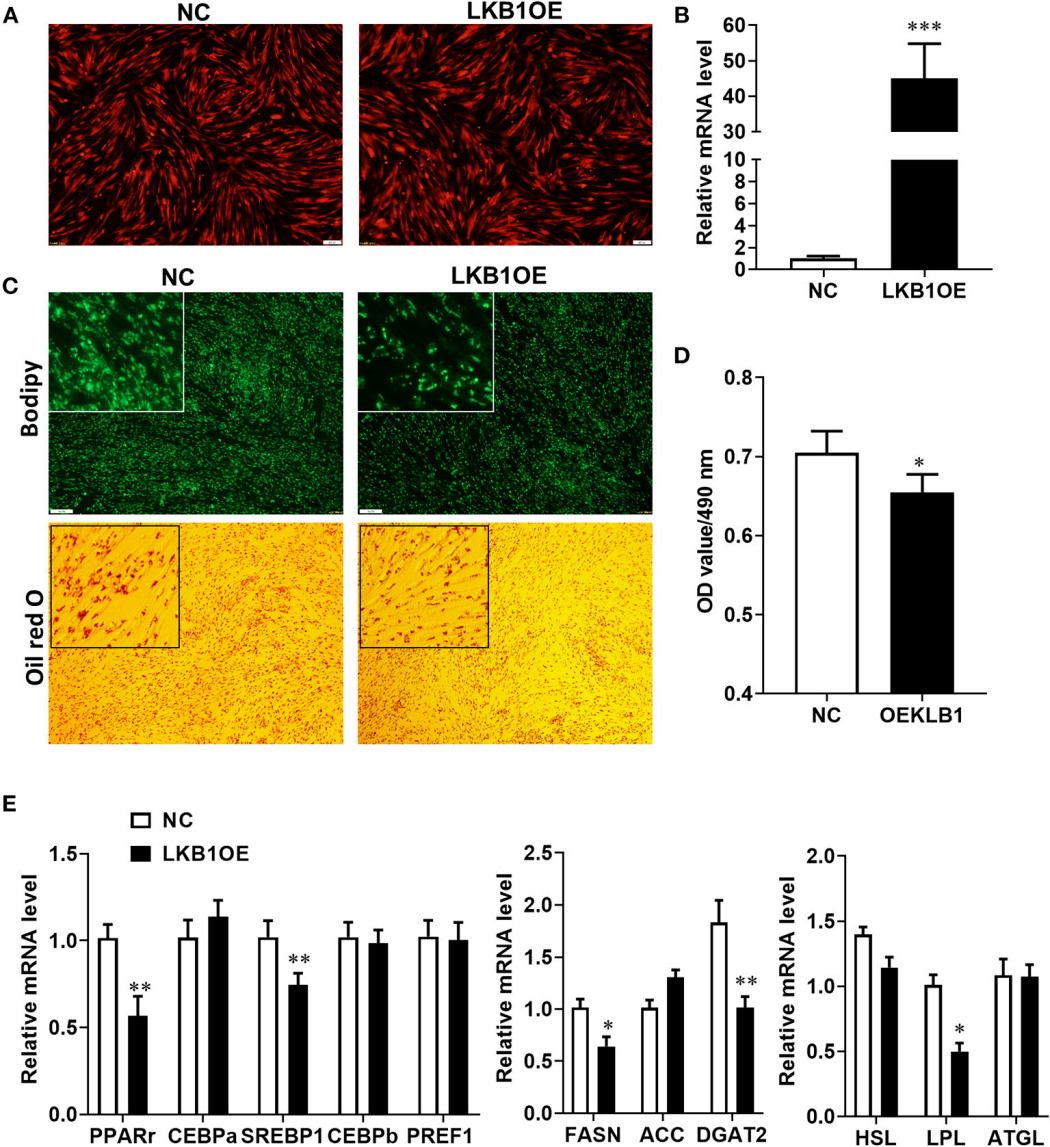
Overexpression of LKB1 inhibits intramuscular adipocyte lipid accumulation and downregulates mRNA level of adipogenic-related genes. **(A)** The images of intramuscular preadipocytes infected by control or overexpressed LKB1 adenovirus. **(B)** The overexpression efficiency of LKB1 in intramuscular adipocyte mediated by adenovirus. **(C)** The images of mature intramuscular adipocytes stained by bodipy and oil red O dye. **(D)** Quantitative analysis of oil red O staining signal was indicated by absorbance at 490 nm. **(E)** The mRNA level of adipogenic transcription factors (*PPAR*γ, *C/EBP*α, *C/EBP*β, and *PREF1*), TG synthesis genes (*FASN, ACC*, and *DGAT2*), and TG lipolysis genes (*HSL, LPL, and ATGL*) between control and LKB1OE groups post-adipogenic induction. *P < 0.05, **P < 0.01, ***P < 0.001. Scale bar: 100 μm. LKB1, liver kinase B1; FASN, fatty acid synthase; ACC, acetyl-CoA carboxylase alpha; DGAT2, diacylglycerol acyltransferase, HSL, hormone-sensitive lipase; LPL, lipoprotein lipase; ATGL, adipose triglyceride lipase.

Our results provide evidence that LKB1 knockdown promotes intramuscular preadipocytes differentiation. Here, we generated adenovirus overexpressing LKB1 and infected goat intramuscular preadipocytes to further clarify the exact role of LKB1 by the gain of function. The images showed that the highly infected efficiency was observed in both control (NC) and LKB1 overexpression (LKB1OE) groups (Figure 5A). The expression of LKB1 was largely upregulated in LKB1OE cells, the elevation of ~40-fold changes expressional level to that of NC with detail (Figure 5B). At the morphology, there were fewer lipid droplets labeled by bodipy dye in the LKB1OE group than that of the control, and a similar phenomenon was observed by oil red O staining (Figure 5C). Statically, the OD value at 490 nm showed that the oil red O signal in the overexpression group was significantly lower than that in the control group (*P*< *0.05*; Figure 5D). In principle, the occurrence of adipogenesis depends on the efficiency of lipid accumulation of preadipocytes differentiating into adipocytes and terminal differentiation, which are regulated by adipogenic transcription factors and genes related to triglyceride synthesis and decomposition (Gulyaeva et al., 2019). Therefore, the mRNA levels of these genes were detected by qPCR. The results showed that LKB1 overexpression significantly downregulated the mRNA levels of adipogenic transcription factors, including *PPAR*γ (*P*< *0.01*) and *SREBP1*(*P*< *0.01*). Moreover, LKB1 gain of function also significantly reduced the mRNA levels of TG biogenesis-related genes (*P*< *0.05*), such as *FASN, DGAT2*, and a lipolysis gene, *LPL* (Figure 5E). Altogether, it is concluded that LKB1 overexpression inhibits lipid accumulation in intramuscular adipocytes.

### *LKB1* Loss of Function Affects the *Mrna* Transcript Profiles in Goat Intramuscular Adipocytes

**Figure 6:**
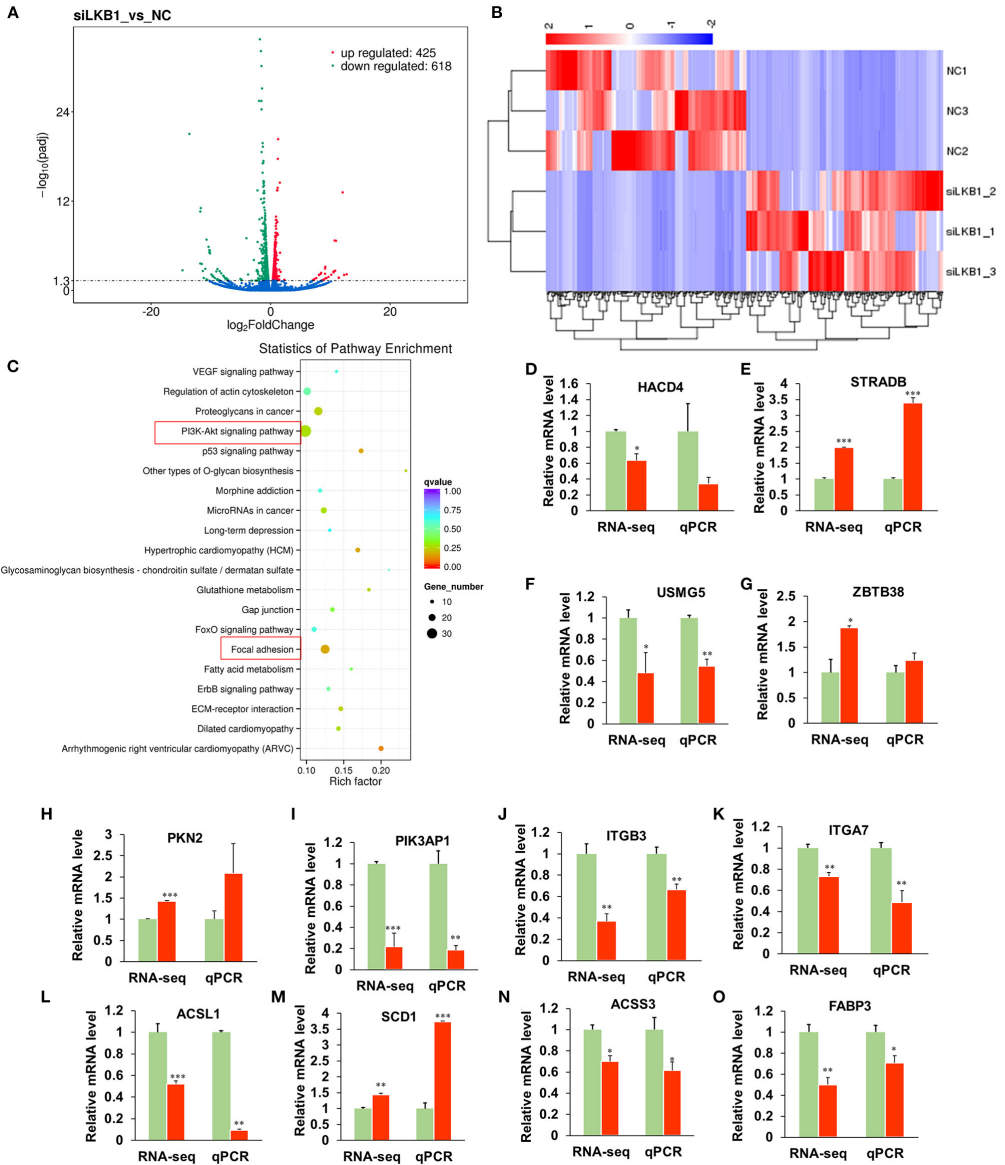
Knockdown of LKB1 affects the mRNA transcript profiles. **(A)** RNA-seq volcano plot of significantly differential expression genes (DEGs) in siLKB1 (*n* = 3) vs. NC (*n* = 3) goat intramuscular adipocytes. Red and blue dots denote upregulated and downregulated genes, respectively. *P* < 0.05 is indicated by the gray dashed horizontal lines. **(B)** The heat map showing the relative levels (fold changes = siLKB1 signal/NC signal) of DEGs. **(C)** The KEGG pathway analysis of related DEGs. **(D–O)** Verification of representative DEGs by qPCR (*n* = 3), including 3-hydroxyacyl-CoA dehydratase 4 (HACD4), STE20-related adaptor beta (STRAD), upregulated during skeletal muscle growth protein 5 (USMG5), Zinc finger and BTB domain containing 38 (ZBTB38), protein kinase N2 (PKN2), phosphoinositide-3-kinase adaptor protein 1 (PIK3AP1), integrin subunit beta 3 (ITGB3), integrin subunit alpha 7 (ITGA7), acyl-CoA synthetase long-chain family member 1 (ACSL1), stearoyl-CoA Desaturase 1 (SCD1), acyl-CoA synthetase short-chain family member 3 (ACSS3), fatty acid-binding protein 3 (FABP3). *P < 0.05, **P < 0.01, ***P < 0.001. LKB1, liver kinase B1; NC, negative control.

To elucidate the underlying molecular mechanism of the LKB1 regulating intramuscular adipogenesis. The total RNAs from NC and LKB1 knockdown intramuscular adipocytes were performed RNA-seq analysis to identify their altered transcriptional profiles. Differentially expressed genes (DEGs) were screened based on the criteria of *p*_*adj*_< *0.05* and *fold change* >*2*. Volcano plots showed a broad overview of changes in gene expression between NC and LKB1 knockdown groups (Figure 6A). Clearly, a strong transcriptional response was observed in LKB1 knockdown intramuscular adipocytes, exhibiting 1,043 genes with significantly altered expression levels, compared to those of NC. Of the 1,043 genes, 425 genes were upregulated, while 618 genes were downregulated (Figure 6A). Next, the DEGs of each group were analyzed by hierarchical cluster analysis in the form of a heat map to provide the visualization of the whole effect of gene expression change (Figure 6B). Subsequently, to further confirm the potential function of DEGs in the intramuscular adipogenesis effect by LKB1 and KEGG pathway analyses were performed. The top 20 of significant difference signaling pathways are shown in Figure 6C, such as arrhythmogenic right ventricular cardiomyopathy (ARVC), focal adhesion pathway, PI3K-Akt signaling pathway, fatty acid metabolism pathway, ErbB signaling pathway, regulation of actin cytoskeleton, VEGF signaling pathway, FoxO signaling pathway, and others (Figure 6C). Of them, PI3K-Akt signaling pathway and focal adhesion had the top 2 enrichment, with 35- and 26-DEGs enriched in these pathways, respectively (Figure 6C), suggesting that the two pathways may be associated with the IMF deposition effect by LKB1. Furthermore, 12 genes were randomly selected from DEGs for validation using qPCR analysis, and all genes we examined showed the same expression trend as observed by RNA-seq, with 10 of them reaching statistical significance (Figure 6D), which confirmed the accuracy of the sequencing data.

### Elevation of Intramuscular Adipocyte Lipid Accumulation Induced by *LKB1* Partial Depends on Focal Adhesion Pathway

**Figure 7:**
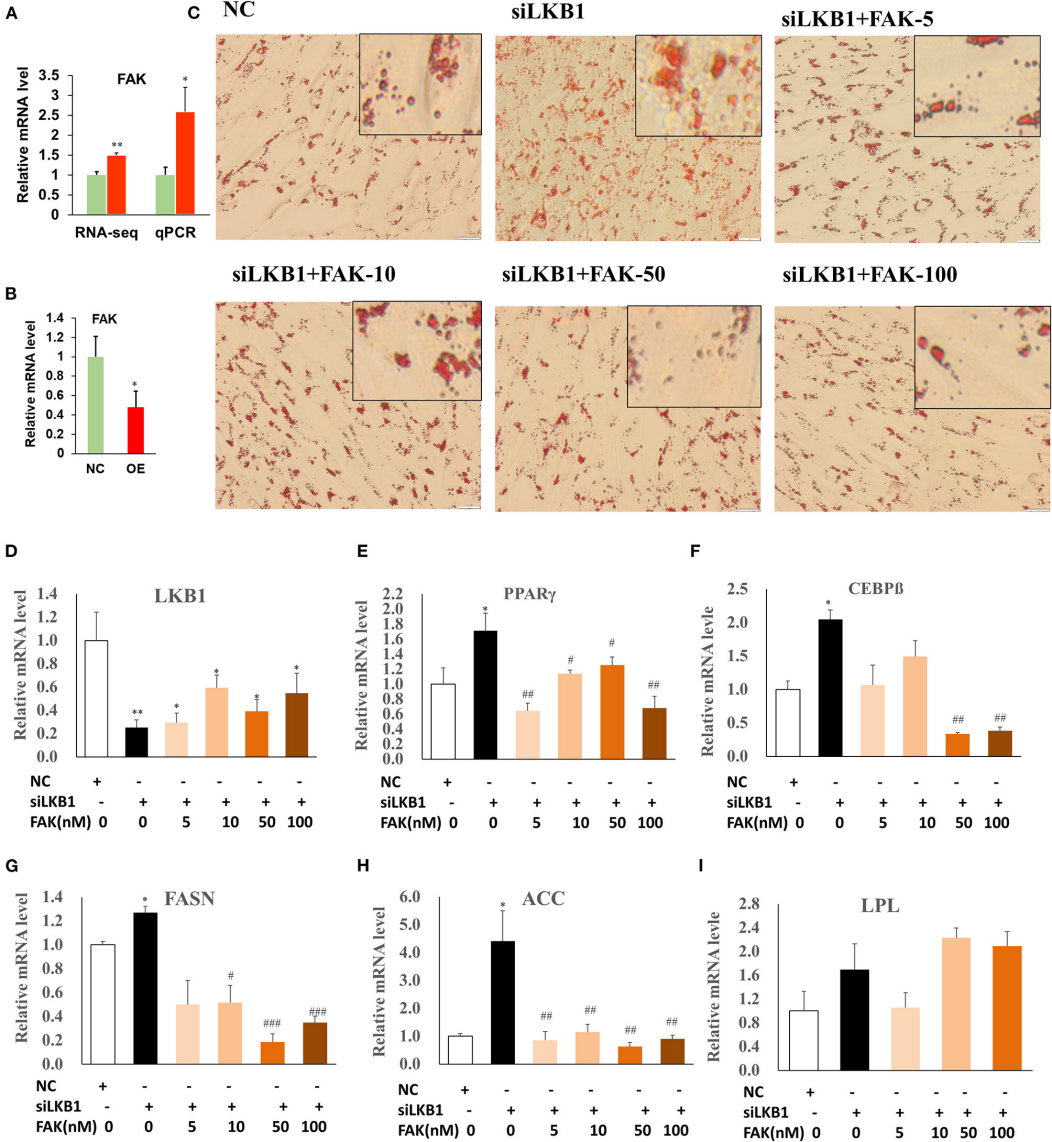
Knockdown of LKB1 promotes intramuscular adipocyte lipid accumulation through FAK pathway. **(A,B)** The mRNA level of FAK gene in siRNA treatment **(A)** and LKB1 overexpressed **(B)** cells by qPCR analysis. **(C)** The oil red O staining images of intramuscular adipocyte cells in NC, siLKB1, and siLKB1 combined with different concentrations of FAK inhibitor (PF573228) treatment groups. **(D–I)** The mRNA level of LKB1 **(D)**, PPAR γ **(E)**, C/EBPβ **(F)**, FASN **(G)**, ACC **(H)**, LPL **(I)** cells in NC, siLKB1, and siLKB1 combined with different concentrations of FAK inhibitor (PF573228) treatment intramuscular adipocyte cells. N = 6. *P < 0.05, **P < 0.01, compared to those of NC group; #*P* < 0.05, ## *P* < 0.01, ### *P* < 0.001, compared to those of siLKB1 group. LKB1, liver kinase B1; FAK, focal adhesion kinase; NC, negative control. FASN, fatty acid synthase; ACC, acetyl-CoA carboxylase alpha; LPL, lipoprotein lipase.

Our RNA-seq data showed that the DEGs were enriched in the focal adhesion pathway, and FAK is one of the main components of the focal adhesion complex to activate this pathway (Tapial Martinez et al., 2020). The expression level of *FAK* was significantly upregulated in the siRNA treatment by both RNA-seq and qPCR analysis, compared to those of control (Figure 7A). Comparatively, overexpression of LKB1 dramatically downregulated the expression level of FAK (Figure 7B). Hypothetically, the promotion of intramuscular adipogenesis mediated by LKB1 knockdown might be through enhancement of focal adhesion pathway. Previous research reported that PF-573228 was a specific inhibitor for FAK (Bai et al., 2021), which was used to determine whether inhibition of the FAK pathway rescues the effect of LKB1 knockdown in goat intramuscular adipocytes. Thus, different concentrations (5, 10, 50, and 100 nM) were set for the rescue experiments, and the data showed that these concentrations did not affect cell viability (Figure S1). The images stained by oil red O showed that siRNA combined with 5 nM FAK inhibitor treatment has less oil red O labeled lipid droplets than that of only siRNA transfected adipocytes (Figures 7C,D). Moreover, this reduction extent became larger and larger at a concentration of 10, 50, and 100 nM and exhibited the dose-dependent manner. In accordance with the above phenotype, the FAK inhibitor rescued all the mRNA levels of detected genes, including adipogenic transcription factor (*PPAR*γ and *C/EBP*β, Figures 7E,F), TG synthesis-related genes (*FASN* and *ACC*, Figures 7G,H), except for *LPL* (Figure 7I). Taken together, these data indicated that LKB1 loss of function induced enhancement of intramuscular adipocyte lipid accumulation at least partially depends on the focal adhesion pathway.

## Discussion

In this study, the goat LKB1 nucleotide sequence was cloned, and expression patterns in various tissues and during intramuscular adipogenesis were revealed. Functionally, we investigated the exact role of LKB1 regulation on goat IMF deposition *in vitro* by loss of and gain of function. Mechanistically, the altered transcript profiles affected by LKB1 knockdown were constructed by RNA-seq and found that knockdown of LKB1 enhanced the goat IMF deposition through the focal adhesion pathway. Overall, this work elucidates the specific molecular mechanism of LKB1 regulating the differentiation of intramuscular adipocytes and provides theoretical support for improving human health and meat quality from the aspect of IMF deposition.

The goat LKB1 gene was cloned, and its tissue expression characteristics were elucidated. It was found that the LKB1 gene was widely expressed in goat various tissues, and the highest expression level was measured in the kidney. Two pieces of evidence might account for this phenomenon. First, the kidney as one of the important and high-energy demanding organs of the body ensures normal metabolism of the organism by removing the poisons and wastes in the body (Han et al., 2016), and LKB1/AMPK pathway was regarded as the metabolic sensor in energy metabolism (Jansen et al., 2009; Han et al., 2016). Second, previous studies reported that epithelial-specific Lkb1 deletion exhibited progressive kidney disease characterized by flattened dedifferentiated tubule epithelial cells, interstitial matrix accumulation, and dilated cystic-appearing tubules (Han et al., 2016) and indicated that Lkb1 is essential for maintaining the normal physiological function of the kidney. Thus, the highest mRNA level in the kidney was observed among detected tissues.

In this work, we provided pieces of strong evidence that supported this conclusion LKB1 as a negative regulator for goat intramuscular preadipocytes adipogenesis. Knockdown of LKB1 promoted goat intramuscular preadipocytes differentiation, manifested by elevation of bodipy and oil red O signal, enhanced the expression levels of adipogenic transcriptional factors and TG synthesis-related genes. Moreover, overexpression of LKB1 reduced the lipid accumulation and decreased adipogenic and lipid droplets biogenic-related genes. It was known that fat deposition was mainly determined by the hyperplasia and hypertrophy of intramuscular adipocytes, the latter is associated with TG biogenesis and its lipolysis (Haczeyni et al., 2018). However, it seems contradictory observation is that promotion of lipid accumulation in LKB1 is also accompanied with upregulation of mRNA level of LPL, a key lipolytic gene, while this gene is downregulated by a gain of LKB1 function. The adipocyte lipid accumulation is a gross effect of the imbalance of TG synthesis and lipolysis (Ducharme and Bickel, 2008; Saponaro et al., 2015). Thus, this might be explained by the role of TG synthesis is greater than that of lipolysis influenced by LKB1 knockdown and the total response is exhibiting promotion of lipid accumulation in this case.

The genome-wide altered transcriptional profiles were constructed by RNA-seq, and 1,043 DEGs were screened in loss of LKB1 intramuscular adipocytes, to uncover the underlying specific molecular mechanism. Interestingly, the PI3K-Akt signaling pathway and the focal adhesion pathway were analyzed as the top two enrichment functional pathways by the KEGG analysis of DEGs. Previous extensive research reported that PI3K/Akt is the classic downstream molecules of FAK signaling (Gao et al., 2018; Zhang et al., 2019b; Wang et al., 2020) and we, therefore, focused on the Focal adhesion pathway as the direct candidate downstream of LKB1. Consistently, knockdown of LKB1 upregulated expression level of FAK, while overexpression of LKB1 downregulated its level. Similarly, it was reported that LKB1 deficiency promotes proliferation and invasion of glioblastoma through activation of focal adhesion kinase signaling pathways (Zhang et al., 2019a). In addition, Kline et al. concluded that LKB1 serves as a FAK repressor to stabilize focal adhesion sites by protein-protein interaction in tumor cells, and when LKB1 function is compromised, aberrant FAK signaling ensues, resulting in rapid FAK site maturation and poor directional persistence (Kline et al., 2013). In intramuscular adipocyte, whether LKB1 direct associates in a complex with FAK or regulated FAK mRNA level through indirect molecules need be further explored.

In intramuscular adipocytes, interference of LKB1 activated the focal adhesion pathway and subsequently led to enhancing the intramuscular adipogenic differentiation. Intriguingly, FAK inhibitor treatment of the siRNA groups rescued the phenotype caused by the decrease of LKB1 expression in the manner of dose-dependent, manifested by debasement of oil red O signal and adipogenic-related genes expression. Thus, we concluded that loss of LKB1 function induced enhancement of intramuscular adipogenic differentiation at least partially depends on the focal adhesion pathway. Supporting our interpretation, previous studies found that FAK signaling is essential in adipocyte differentiation (Li and Xie, 2007; Yuan et al., 2018). In detail, preadipocytes differentiated into the physiological function of adipocytes are characterized by major cell morphology from fibroblastic to a rounded shape, which is closely associated with alterations in cytoskeleton and cell–ECM contacts (O'Shea Alvarez, 1991). FAK signaling pathway was proved to playing an important role in the both above biological processes (Nagamatsu et al., 2017; Zhao et al., 2018; Tapial Martinez et al., 2020). Thus, we speculated that LKB1 regulated the focal adhesion pathway and subsequently at least partially affect preadipocyte differentiation through altering the morphology of cells.

In conclusion, our studies validate LKB1 as the key negative factor for goat intramuscular preadipocytes differentiation and reveal LKB1 regulation on intramuscular adipogenesis through the focal adhesion pathway. These findings contribute to expand the molecular regulation network on IMF deposition and provide theoretical support for improving human health and meat quality from this aspect.

## Data Availability Statement

The datasets presented in this study can be found in online repositories. The names of the repository/repositories and accession number(s) can be found below: NCBI Sequence Read Archive and Bioproject number PRJNA761512.

## Ethics Statement

The animal study was reviewed and approved by Institutional Animal Care and Use Committee, Southwest Minzu University.

## Author Contributions

YX and YL conceived the project and designed the research. YX, YW, and QX performed all the experiments and analyzed the data. AL, QY, and YM cultured the cells. YX and YW wrote the manuscript. All authors contributed to the article and approved the submitted version.

## Funding

This work was funded by the National Natural Sciences Foundation of China under Grants (Numbers 31902154 and 32072723); the Applied Basic Research Program of Sichuan Province under Grant (Number 2019JY0258); and Fundamental Research Funds for the Central Universities, Southwest Minzu University under Grant (Number 2021112).

## Conflict of Interest

The authors declare that the research was conducted in the absence of any commercial or financial relationships that could be construed as a potential conflict of interest.

## Publisher's Note

All claims expressed in this article are solely those of the authors and do not necessarily represent those of their affiliated organizations, or those of the publisher, the editors and the reviewers. Any product that may be evaluated in this article, or claim that may be made by its manufacturer, is not guaranteed or endorsed by the publisher.
