# Supplementary material for: LKB1 Regulates Goat Intramuscular Adipogenesis Through Focal Adhesion Pathway
Source: Front Physiol. 2021 Oct 13;12:755598. doi: 10.3389/fphys.2021.755598 (PMC8548615; doi:10.3389/fphys.2021.755598)
Supplement: Supplementary file 1 [file Data_Sheet_1.docx]

Figure S1. The effect of FAK inhibitor (PF-573228) on intramuscular adipocytes viability by MTT assay at 24 h and 48 h after PF-573228 (5, 10, 50 and 100 nM) or DMSO treatment. N=8.

**Table S1 The primer sequence of qPCR analysis**

| Gene | Sequence(5'-3') | Sequence(5'-3') | Tm/℃ | GenBank | application |
| --- | --- | --- | --- | --- | --- |
| *LKB1* | ATGGAGGTGGCGGACCCGCA | TCATTGCTGTTTGCAGGCCGACA | 70 | XM_018050463.1 | cloning |
| *LKB1* | AAGCCGTTCTCCATACAGCA | CCTCCAGGTAGGGCATCACT | 60 | XM_018050463.1 | qPCR |
| *PPARγ* | AAGCGTCAGGGTTCCACTATG | GAACCTGATGGCGTTATGAGAC | 60 | NM_001285658.1 | qPCR |
| *C/EBPα* | CCGTGGACAAGAACAGCAAC | AGGCGGTCATTGTCACTGGT | 58 | XM_018062278.1 | qPCR |
| *C/EBPβ* | CAAGAAGACGGTGGACAAGC | AACAAGTTCCGCAGGGTG | 66 | XM_018058020.1 | qPCR |
| *PREF1* | CCGGCTTCATGGATAAGACCT | GCCTCGCACTTGTTGAGGAA | 65 | KP686197.1 | qPCR |
| *FASN* | TGTGCAACTGTGCCCTAG | GTCCTCTGAGCAGCGTGT | 57 | NM_001285629.1 | qPCR |
| *ACC* | GGAGACAAACAGGGACCATT | ATCAGGGACTGCCGAAAC | 60 | XM_018064169.1 | qPCR |
| *DGAT2* | CATGTACACATTCTGCACCGATT | TGACCTCCTGCCACCTTTCT | 60 | BT030532.1 | qPCR |
| *HSL* | AGGGTCATTGCCGACTTCC | GTCTCGTTGCGTTTGTAGTGC | 60 | XM_018062484.1 | qPCR |
| *LPL* | TCCTGGAGTGACGGAATCTGT | GACAGCCAGTCCACCACGAT | 60 | NM_001285607.1 | qPCR |
| *ATGL* | GGTGCCAATATCATCGAGGT | CACACCCGTGGCAGTCAG | 64 | NM_001285739.1 | qPCR |
| *SREBP*1 | AAGTGGTGGGCCTCTCTGA | GCAGGGGTTTCTCGGACT | 58 | NM_001285755.1 | qPCR |
| *HACD4* | GATGCAACTTTGCCAGTCTGT | GACTGAACCATGTCAAGACAGC | 60 | XM_018051907.1 | qPCR |
| *USMG5* | GAACCCTTCAGGCCCGTTG | GCTTCTGGACCTGCCATGAT | 60 | XM_005698422.3 | qPCR |
| *STRADB* | GGAGCAGTGAGAGGGTTGAA | GCTGCACTGATGTGCTGAAC | 60 | XM_018060888.1 | qPCR |
| *ZBTB38* | TGGAGGACTCAGAACCAAGGA | AGCACTGTGTCGCTGTGTAA | 60 | XM_018049757.1 | qPCR |
| *PKN2* | TCTCTCTCCGCTCCTAACCC | GCAATCTGCAGCTCGGTGAG | 60 | [XM_018045702.1](https://www.ncbi.nlm.nih.gov/entrez/viewer.fcgi?db=nucleotide&id=1062964214) | qPCR |
| *PIK3AP1* | GAACCTCACTGCCTTGTTGC | TTCATGAGCAGGTCTGTGGAC | 60 | XM_018041577.1 | qPCR |
| *ITGB3* | CGCTACAAGGGGGAGATGTG | CCAGTCGGAGTCACATAGGC | 60 | XM_018065309.1 | qPCR |
| *ITGA7* | GTTTCCTGTCCTCCGCATGG | CGGAGATGGGAATGGGAGTG | 60 | [XM_018048128.1](https://www.ncbi.nlm.nih.gov/entrez/viewer.fcgi?db=nucleotide&id=1062970736) | qPCR |
| *ACSL1* | TCCTACGGCAGTGATCTCCT | CGAAATGTGTGTGTCACTGGC | 60 | XM_005698718.3 | qPCR |
| *SCD1* | TGGCGTTCCAGAATGACGTT | ACCCCATAGATACCACGGCA | 60 | NM_001285619.1 | qPCR |
| *ACSS3* | AGCAATCCGTCAACAGGACC | TCCAGTCTCAGTTTGCCACC | 60 | XM_018047605.1 | qPCR |
| *FABP3* | ATGAAGTCACTCGGTGTCGG | GTCATCTGCCGTGGTCTCAT | 60 | XM_013971605.2 | qPCR |
| *UXT* | GCAAGTGGATTTGGGCTGTAAC | ATGGAGTCCTTGGTGAGGTTGT | 60 | XM_005700842.2 | qPCR |
| *RPLPO* | TCCAGGCTTTAGGCATCACC | AGCACTTCGGGGTTGTAGATG | 56 | XM_005709526.3 | qPCR |
| *PPIA* | ACAAAGTCCCGAAGACAGCAG | AAGTCACCACCCTGGCACAT | 59 | XM_018047035.1 | qPCR |
| *PPIB* | ACACCAACGGCTCCCAGT | AGGCTTGTCCCGACCATC | 60 | XM_005685667.3 | qPCR |
| *FAK* | TTGTGCACAGGGACATTGCT | ATGCGCCCGATCACATCATT | 60 | XM_018058634.1 | qPCR |
